# Fine-Tuning of PI3K/AKT Signalling by the Tumour Suppressor PTEN Is Required for Maintenance of Flight Muscle Function and Mitochondrial Integrity in Ageing Adult *Drosophila melanogaster*

**DOI:** 10.1371/journal.pone.0143818

**Published:** 2015-11-23

**Authors:** Lawrence B. Mensah, Claire Davison, Shih-Jung Fan, John F. Morris, Deborah C. I. Goberdhan, Clive Wilson

**Affiliations:** Department of Physiology, Anatomy and Genetics, University of Oxford, Le Gros Clark Building, South Parks Road, Oxford, OX1 3QX, United Kingdom; National University of Singapore, SINGAPORE

## Abstract

Insulin/insulin-like growth factor signalling (IIS), acting primarily through the PI3-kinase (PI3K)/AKT kinase signalling cassette, plays key evolutionarily conserved regulatory roles in nutrient homeostasis, growth, ageing and longevity. The dysfunction of this pathway has been linked to several age-related human diseases including cancer, Type 2 diabetes and neurodegenerative disorders. However, it remains unclear whether minor defects in IIS can independently induce the age-dependent functional decline in cells that accompany some of these diseases or whether IIS alters the sensitivity to other aberrant signalling. We identified a novel hypomorphic allele of PI3K’s direct antagonist, *Phosphatase and tensin homologue on chromosome 10* (*Pten*), in the fruit fly, *Drosophila melanogaster*. Adults carrying combinations of this allele, *Pten* ^5^, combined with strong loss-of-function *Pten* mutations exhibit subtle or no increase in mass, but are highly susceptible to a wide range of stresses. They also exhibit dramatic upregulation of the oxidative stress response gene, *GstD1*, and a progressive loss of motor function that ultimately leads to defects in climbing and flight ability. The latter phenotype is associated with mitochondrial disruption in indirect flight muscles, although overall muscle structure appears to be maintained. We show that the phenotype is partially rescued by muscle-specific expression of the Bcl-2 homologue Buffy, which in flies, maintains mitochondrial integrity, modulates energy homeostasis and suppresses cell death. The flightless phenotype is also suppressed by mutations in downstream IIS signalling components, including those in the mechanistic Target of Rapamycin Complex 1 (mTORC1) pathway, suggesting that elevated IIS is responsible for functional decline in flight muscle. Our data demonstrate that IIS levels must be precisely regulated by *Pten* in adults to maintain the function of the highly metabolically active indirect flight muscles, offering a new system to study the *in vivo* roles of IIS in the maintenance of mitochondrial integrity and adult ageing.

## Introduction

The insulin/insulin-like growth factor signalling (IIS) cascade and one of its major target pathways involving the nutrient-sensitive kinase complex mTORC1 (mechanistic Target of Rapamycin Complex 1) play key evolutionarily conserved roles in nutrient homeostasis, cell growth regulation, autophagy and longevity [1]. The dysfunction of these pathways is associated with several human diseases including diabetes, cancer and neurodegenerative disorders, though in the latter case, the mechanisms involved have not been fully elucidated.

When insulin and insulin-like molecules bind to receptor tyrosine kinases at the surface of cells, they activate a kinase cascade involving the Class I lipid kinase PI3-kinase (PI3K) and the downstream protein kinase, Akt. Akt has numerous target proteins, but genetic studies in the fruit fly, *Drosophila melanogaster*, have been especially useful in highlighting indirect regulation of the mTORC1 complex as a key step in the control of cell growth and lifespan [2]. Subsequent experiments in mice have suggested that these functions are evolutionarily conserved [3,4]. The effects of IIS/mTORC1 on lifespan were suggested to be partly connected to the life-extending actions of caloric restriction, though amino acid balance may be a more critical factor [5]. Hypomorphic mutations in positive regulators of IIS signalling are long-lived in flies and mice [6], suggesting that changes in signalling alone are sufficient to alter the ageing process. However, the IIS-regulated cellular changes that modulate functional decline of tissues like nerve and muscle during ageing are not fully understood.

Other genetic screens in flies have also suggested that reducing mTORC1 signalling in models of neurodegeneration suppresses the disease phenotype [7,8]. For example, the degenerative phenotype produced by expression of the Huntington protein in the developing fly eye is reduced by mutations that decrease mTORC1 signalling via a mechanism that involves induction of autophagy [9]. Decreased mTORC1 signalling also suppresses degeneration in fly brains overexpressing Tau, a model for Alzheimer’s Disease, though in this case, inhibition of cell cycle activator expression was highlighted as an important suppression mechanism [7].

There are indications that altered IIS activity is associated with degenerative processes in humans, but both increased and reduced signalling have been implicated [10], perhaps reflecting the complex range of mechanisms involved. It therefore remains unclear whether increased IIS could normally promote age-dependent degeneration in the absence of other sensitising factors or whether IIS-induced functional decline with age can take place without overt cell degeneration. One study in flies has demonstrated that high level overexpression of the mTORC1 activator Rheb induces muscle degeneration in adults [11]. However, the Rheb transgene employed produces a strong upregulation of mTORC1 activity and induces substantial overgrowth in other tissues [12], making it difficult to determine the physiological relevance of these data.

Here we describe the phenotypic characterisation of a hypomorphic allele of *Pten* in adult flies. The evolutionarily conserved PTEN protein is a lipid phosphatase that directly antagonises the lipid kinase activity of PI3K, and therefore reduces IIS activity [13,14]. We show that when this allele and much stronger *Pten* loss-of-function alleles are combined, the transheterozygous flies exhibit little or no increase in size, but are more susceptible to a variety of stresses. Importantly, they progressively become flightless and exhibit other motor defects with age. Our genetic and cell biological data indicate that this phenotype is caused by increased IIS/mTORC1 activity, which progressively affects indirect flight muscle function. We show that although overall muscle structure is maintained, the mitochondria in these cells are severely disrupted, indicating that subtle elevation of IIS can selectively affect mitochondrial integrity and cell function in this highly metabolically active tissue.

## Materials and Methods

### *Drosophila* stocks

*CantonS*, *w* ^*1118*^,*4E-BP* (*Thor* ^*2*^) [15], *how-GAL4* [16], *arm-GAL4*, and *UAS-buffy* [17] stocks were obtained from the Bloomington Stock Centre. Flies used in this study carry alleles including; *Pten* ^*1*^ and *Pten* ^*3*^ [13,18], *Pten* ^*dj189*^ and a *Pten* genomic transgene [14], *foxo* ^*25c*^ and *foxo* ^*21a*^ [19,20], Akt1^q^ [21] and *Akt1* ^*3*^ [22], *Tor* ^*ΔP*^ [23] and *Rheb* ^AV4^ [24]. The *Pten* ^*5*^ allele was generated in the screen reported in [13] and like *Pten* ^*3*^, is located on an otherwise non-mutant chromosome carrying the *P[w* ^*+*^ *]30C P[ry* ^*+*^, *neo-FRT]40A* transgenes.

### Molecular analysis of the *Pten* ^*5*^ allele

DNA was extracted from adult females transheterozygous for the *Pten* ^*5*^ allele and the *Df(2L)170B* chromosome, in which the entire *Pten* gene is deleted [13]. The *Pten* ^*5*^ chromosome was sequenced using the following 8 pairs of primers: Pten 322F: ATAGAAGACAAGCACTGGTTC and Pten 719R: CGCTCCGAGCATAGGTTATAG; Pten 629F: GCCTATTCAGAAACCGTCTGG and Pten 957R: GTTCTGCCCTTTCCAGCTTTAC; Pten 913F: GTCCAATGTTGTAGCCGTGC and Pten 1367R: CACACAACTGGACTCCGAGAAG; Pten 1252F: AGCCTTAACGTGAGTATTTCCAGC and Pten 1668R: ATCGCCGGAAACTGGTATTGATG; Pten 1402F: TATTACACGACTCAGCCACAG and Pten 1830R: CCATCGGACTCGCAAGCTAAAG; Pten 1808F: TCTTTAGCTTGCGAGTCCGATG and Pten 2345R: CTATTAGGCTGTTTGCGTTTGCAC; Pten 2308F: AATACTTCGACTGCGTGCAAAC and Pten 2628R: CTGGTCATTGAGAGTATAGTGTGC; Pten 3200F: CACTGCCATTGTCCTTCTACTC and Pten 3603R: TCATACAGTATATTTACAAATTCGAA.

### Light microscopy and eye phenotype image analysis

To photograph and analyse fly eyes, a Leica Wild M35 stereomicroscope was used with an Axiocam digital camera. Defective eye structure was scored on the basis of roughness or disorganisation of the usually perfectly hexagonal arrangement of the ommatidia. The image analysis programme, Axiovision was employed to capture the eye phenotype and Adobe PhotoShop CS4, was used to process digital images.

### Body Mass Assay

Body mass was determined as described previously [25,26]. For each genotype, batches of five one-day-old female and male flies were weighed. The weighing of each batch was performed three times for at least ten groups of flies from two independent genetic crosses.

### Flight Assay

The flight assay was performed as described previously [27,28] with minor modifications. Female and male flies were collected in separate vials shortly after eclosion, and maintained in a 25°C incubator for 1–2 days with a 12 h light-dark cycle on standard cornmeal food (10.5g of technical grade agar, 75.0g of cornmeal, 31.5g of dried yeast, 93.0g of glucose, 8.6g of sodium potassium tartrate, 0.7g of calcium chloride and 2.5g of methyl-4-hydrobenzoate [nipagen] per litre). Flies were tested for early-onset of flightlessness on day 2. For flight tests in older flies, flies were transferred every three days onto fresh food, maintained at 25°C and tested at the required time point. They were allowed to acclimatise for 1–2 h at room temperature prior to flight testing [27,28]. Batches of five flies, sorted by tapping flies between vials (and not by anaesthetizing with CO_2_), were transferred into a petri dish, which was quickly covered with the lid. The petri dish was held at a height of one meter, inverted and gently tapped to release flies. The numbers of flies that flew away, fell vertically or veered to the side were recorded. Those that veered to the side or fell were retested to confirm the flightless phenotype.

### Climbing

A geotaxis and motor function test was performed as described previously [28,29] with minor modifications. Nine-day-old male flies were moved to ambient temperature for 1–2 h for acclimatisation [28]. For each genotype, climbing ability was assessed for five different groups of 10 flies. Each group of flies was transferred into a graded cylinder with a climbing height marked at 6cm. After tapping flies to the bottom of the cylinder, flies were given 30 seconds to climb to or pass the 6cm mark. The number of flies that failed to reach the 6cm mark was recorded. These trials were repeated five times for each group. The average number of flies that failed to reach the 6cm mark was calculated for each genotype as described in [28,29], and was expressed as a percentage (mean % ± s.d). Statistical significance was assessed using one-way ANOVA with Bonferroni post-hoc correction.

### Water-only dietary restriction assay

Water-only starvation was performed as described previously [30] with minor modifications. At least seven groups of twenty 1–2 day old males were anaesthetized and transferred onto a water-soaked cotton ball placed in the bottom of a vial. Vials were placed in a tray containing water to prevent gradual desiccation of the moist-cotton ball and incubated at 25°C. Every 24 h, 2–3 drops of water were added to the cotton wool balls to keep them moist at all times. Six to eight vials were assayed simultaneously for each genotype and the number of viable flies (mean ± s.d) calculated.

### Stress assays

Fly food was prepared as described previously [27]. Groups of twenty 1-2-day-old males were collected into separate vials and maintained in a moist chamber at 25°C to prevent the food from desiccation. Flies were transferred onto fresh food every three days. Typical food for the stress assays consisted of 0.8% agar and 5% sucrose in combination with the specific inducer compound. For the osmotic stress assay, 500mM NaCl [31] was added. To induce oxidative stress, food containing 2mM paraquat was used [27]. 5mM rotenone [31] was employed to test flies for susceptibility to mitochondrial complex 1 inhibition.

### Fixation and embedding of adult thoraces for light and electron micrograph

The thoraces of anaesthetized adult flies were fixed in 4% paraformaldehyde (Sigma, UK), 2.5% gluteraldehyde (TAAB laboratories, Berkshire, UK) in 0.1M PBS, pH 7.4. Thoraces were washed with 0.1M PBS, pH 7.4 for 3 x 10 min and stained with 1% aqueous Osmium tetroxide (0.1M PBS, pH7.4) for 1–2 h followed by 3 x 10 min washes with distilled water. Osmication was followed by staining in 2% uranyl acetate (dissolved in water), dehydration and subsequent embedding in Spurr’s resin. The Spurr’s resin block was cut at 1μm thickness, stained with 1% toluidine blue in 1% borax and examined using the light microscope to check tissue orientation and quality of sectioning. Ultra-thin sections of 60nm were made on an Ultracut E microtome and collected on copper grids. The sections were stained with 5% uranyl acetate for 30min followed by 4min incubation with Reynolds lead citrate prior to examination with a Jeol JEM-1010 transmission electron microscope (Jeol LTD, Hert, UK).

### Quantitative RT-PCR

All larvae were staged to wandering third instar and the genotype was distinguished as homozygous mutant (test) and heterozygous (control) by absence or presence of the dominant *Tubby* balancer phenotype respectively. Tissues were preserved in 400μl RNA*later* RNA stabilizing buffer (Qiagen, UK). Total RNA was extracted from 15 third instar larvae in three independent experiments using RNeasy mini RNA extraction kit (Qiagen, UK). Final RNA concentration was determined spectrophotometrically at 260 and 280 nm on an ND-1000 NanoDrop (Thermo Fisher Scientific, UK). For cDNA synthesis, 50 ng of total RNA in 20 μl cDNA synthesis mastermix was used to reverse-transcribe RNA to cDNA using the High Capacity Reverse Transcription kit (Applied Biosystems, UK) on a Gene Amp PCR system 9700 machine (Applied Biosystems, UK). Real-time quantitative PCR was carried out with qRT-PCR SYBR Green premix thermostable Taq polymerase and MgCl kit (Primer Design Ltd, UK) on a 7000 ABI Prism platform (Applied Biosystems, UK). All reactions were performed in triplicate using the standard curve method. Melting curves were examined after amplifications to confirm single-product measurement. Transcript levels were normalized to the *Ribosomal protein L32* (*RpL32*) housekeeping gene transcript level. All final values were normalized to the transcript level in *w* ^*1118*^ flies in order to determine the gene expression fold-change in relation to normal animals. The following *Drosophila*-specific quantiTech primers used for these assays were purchased from Qiagen: *Ribosomal protein L32*, Entrez Gene ID 43573, QuantiTect Primer ID QT00985677; *Pink1*: PTEN-induced putative kinase 1, Entrez Gene ID 31607, QuantiTect Primer ID QT00499380; *GstD1*, Glutathione S transferase D, Entrez Gene ID 141503, QuantiTect Primer ID QT01170659.

### Statistical Analyses

Eye phenotype and body mass were statistically analysed using a two-tailed unpaired Student’s *t-test*. Two-way Analysis of Variance (ANOVA) with a Bonferroni post-hoc correction test for multiple comparisons was used for analysis of the flightless phenotype at multiple ages. One-way ANOVA with a Bonferroni post-hoc correction test was used to analyse single time-point flightless phenotypes and genomic rescue experiments, climbing activity and transcript expression levels. Unless otherwise stated, mean values represent four or more independent experiments, involving at least two independent replicates and at least two independent experiments. Statistical analyses were performed with the statistical programme, GraphPad Prism (Version 5.01, GraphPad Software, La Jolla, CA, USA).

## Results

### Transheterozygous hypomorphic *Pten* mutants exhibit a highly specific and penetrant recessive eye phenotype

The novel recessive viable *Pten* mutant, *Pten* ^*5*^, was isolated from a screen for mutations that failed to complement the deficiency, *Df(2L)170B*, which uncovers *Pten* [13]. It was identified because flies transheterozygous for *Pten* ^*5*^ and *Df(2L)170B* had eyes with a slightly disorganised ommatidial array. Homozygous *Pten* ^*5*^ flies, which also displayed a rough eye phenotype, rarely eclosed, suggesting that there was a second mutation on this chromosome that increased lethality. However, we were unable to remove this mutation in subsequent recombination experiments, and instead worked with several viable transheterozygous *Pten* ^*5*^ combinations (see below).

**Fig 1:**
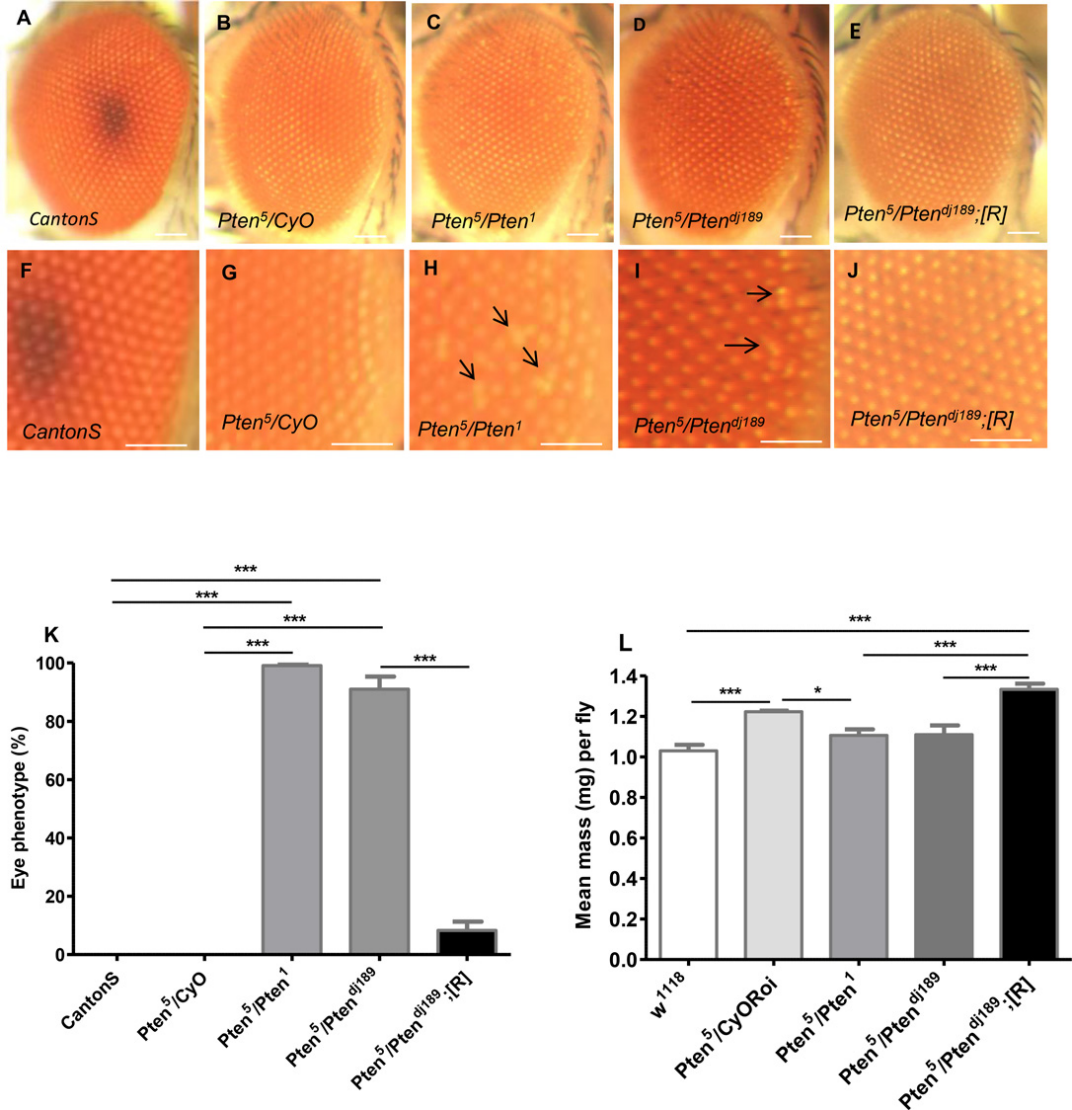
Transheterozygous *Pten* ^*5*^ mutant flies have a highly penetrant eye phenotype. **A-J**. Low (**A-E**) and high (**F-J**) magnification views of eyes from females of different genotypes. A mild disorganisation of the ommatidia in the posterior region of the eye is observed in *Pten* ^*5*^ */Pten* ^*1*^ (**C**,**H**) and *Pten* ^*5*^ */Pten* ^*dj189*^ (**D**, **I**) flies, as shown by black arrows in **H** and **I**, but not in wild type *CantonS* (**A**,**F**) and *Pten* ^*5*^ heterozygous control females (*Pten* ^*5*^ */CyO*) (**B**,**G**). Almost all female mutant animals carrying a *Pten* genomic rescue construct (*Pten* ^*5*^ */Pten* ^*dj189*^ *;[R]*) (**E**,**J**) do not display the eye phenotype. (**K**) Histogram presented as mean percentage of flies exhibiting disorganised eye phenotype. Error bars indicate standard error of mean (SEM). *** *P* < 0.001, from two separate experiments n ≥ 100. (**L**) The mean body mass of different *Pten* mutant females, *Pten* ^*5*^ */Pten* ^*1*^ and *Pten* ^*5*^ */Pten* ^*dj189*^, is not significantly heavier than wild type *w* ^*1118*^. Surprisingly, *Pten* ^*5*^ */Pten* ^*dj189*^ rescue females have significantly higher body mass than all other genotypes. Data are presented as mean body mass per fly ± SEM. Pooled from two independent experiments, n ≥60. Statistical significance was determined by two-tailed unpaired Student’s *t*-test. Scale bar: 100μm.

To determine whether the eye phenotype was associated with *Pten* loss-of-function, we combined *Pten* ^*5*^ with two independently derived *Pten* alleles that have previously been shown to behave like nulls, *Pten* ^*1*^ [13] and *Pten* ^*dj189*^ [14]. *Pten* ^*1*^ has not been molecularly characterised, while *Pten* ^*dj189*^ contains an F-element insertion in the *Pten* coding sequence after amino acid 89, which should remove the phosphatase domain of the protein. Both mutant combinations were viable and exhibited a highly penetrant, but mild, disorganised eye phenotype at the posterior edge in the midline (Fig 1). The phenotype was observed in females in all *Pten* ^*5*^ */Pten* ^*1*^ and 92% of *Pten* ^*5*^ */Pten* ^*dj189*^ flies (*P* < 0.001; Fig 1K), and in males in all and 82% of flies respectively (S1A Fig). It was never observed in heterozygous *Pten* ^*5*^/*CyO* or wild type *CantonS* controls. Importantly, only 9% of *Pten* ^*5*^ */Pten* ^*dj189*^ females carrying a *Pten* genomic rescue construct [14] (Fig 1K) and no male flies of this genotype (S1A Fig) displayed this phenotype, demonstrating that the defect, which probably arises during larval development, is caused by reduced *Pten* activity. Oldham et al. (2002) previously isolated a temperature-sensitive viable hypomorphic allele of *Pten*, *Pten* ^*2L100*^, and reported that flies occasionally had slightly rough eyes [30], perhaps reflecting a similar, but less penetrant defect.

PTEN acts antagonistically to PI3K to suppress cell, tissue and whole body growth [1]. Weighing several small groups of flies, as we and others have done in the past [25,26], did not reveal any significant weight differences in mutant versus non-mutant *Pten* adults. When we analysed more groups, we did see modest differences, but these changes did not indicate that the reduction in *Pten* function associated with viable *Pten* ^*5*^ mutant animals is sufficient to increase growth. For example, transheterozygous *Pten* ^*5*^ */Pten* ^*1*^ and *Pten* ^*5/*^ *Pten* ^*dj189*^ females did not have a significantly greater body mass than heterozygous *Pten* ^*5*^/*CyO Roi*, transheterozygous mutants carrying a genomic rescue construct, or *w* ^*1118*^ controls; in fact some of these latter genotypes were slightly heavier (Fig 1L). By contrast, transheterozygous mutant *Pten* ^*5*^ */Pten* ^*1*^ males did have significantly (~20%) greater body mass than heterozygous *Pten* ^*5*^ */CyoRoi* and wild type *w* ^*1118*^ (S1B Fig), but *Pten* ^*5/*^ *Pten* ^*dj189*^ males did not (S1B Fig). There is no clear explanation for the differences between males and females in these assays, but previous studies have also shown different relative changes in mutant versus control weights between males and females with altered IIS signalling [25]. Overall, our data indicate that there is no consistent *Pten*-dependent effect on fly growth in *Pten* ^*5*^ transheterozygotes. We conclude that genetically *Pten* ^*5*^ is a weak hypomorphic mutation, producing a subtle developmental recessive phenotype in the eye.

To determine the molecular defect associated with the *Pten* ^*5*^ allele, we sequenced the complete *Pten* open reading frame from the mutant chromosome. A single A to G (GAC to GGC) point mutation was identified at position 509, which causes an aspartate (Asp) to glycine (Gly) change at position 170 of the PTEN protein. This residue is highly conserved (Asp162 in human PTEN) and resides in the catalytic TI-loop of the PTEN phosphatase domain. A similar Asp to Ala mutation in the human protein reduces *in vivo* lipid phosphatase activity by approximately 30% [32], consistent with our genetic assignment of this allele as hypomorphic.

### *Pten* ^*5*^ transheterozygous mutants show increased susceptibility to a wide range of stresses

**Fig 2:**
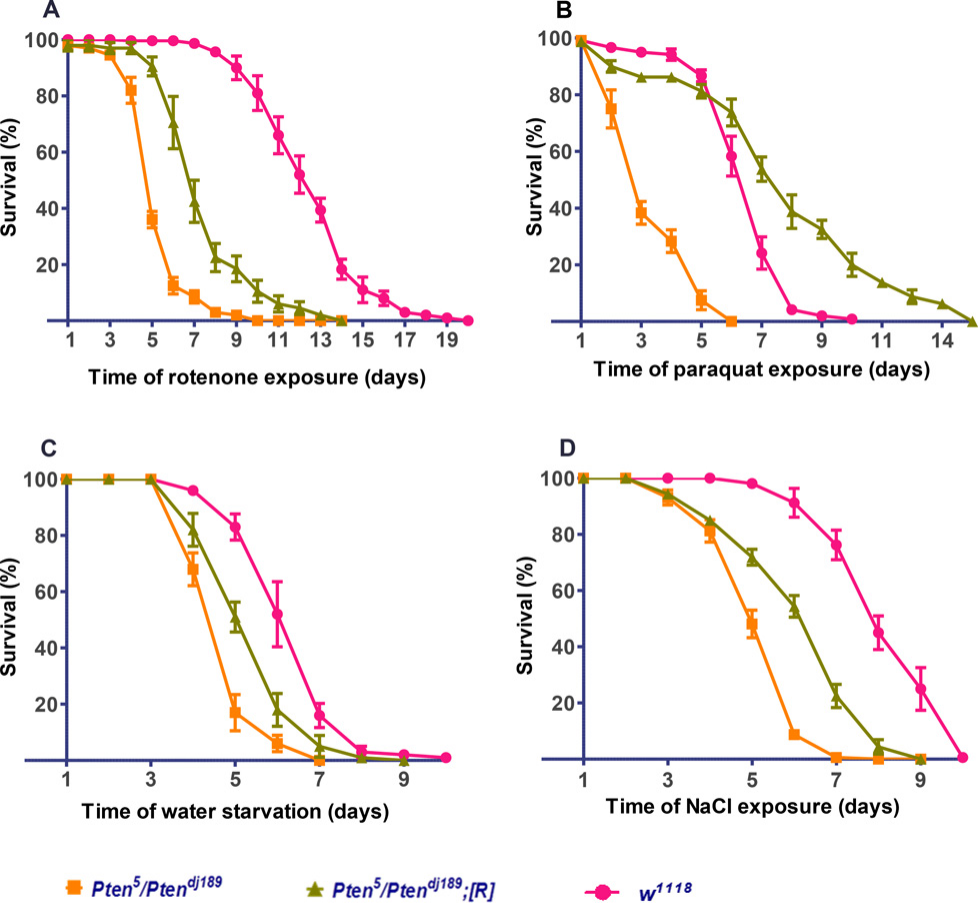
*Pten* ^*5*^ transheterozygous mutant flies are sensitive to a wide range of stresses. Survival of *Pten* ^*5*^ */Pten* ^*dj189*^ transheterozygous mutants (orange), mutants carrying a *Pten* genomic rescue construct (*Pten* ^*5*^ */Pten* ^*dj189*^ *;[R]*) (green), and wild type control *w* ^*1118*^ (pink) males after they were exposed to (**A**) 5mM rotenone, (**B)** 2mM paraquat, (**C)** water-only diet and **(D)** 500mM NaCl. The mean survival times (in hours) for *Pten* ^*5*^ transheterozygous mutants, rescue flies, and wild type control *w* ^*1118*^ flies respectively are: rotenone = 40.4, 89.2 and 107.1; paraquat = 8.6, 24.2, and 50.3; water starvation = 9.2, 19.1 and 24.2; NaCl = 15.4, 19.4 and 30.9. In all four stress assays, *Pten* ^*5*^ transheterozygous mutants were short lived compared with wild type *w* ^*1118*^ (*P* < 0.001), and for all but NaCl stress had a significantly shorter mean survival time compared to rescue flies (*P* < 0.01). For each experiment, flies were grouped into at least 6–8 batches of 20, these experiments were then repeated four times and data pooled together, n ≥ 480). Statistical significance was determined by Mantel-Cox Log rank test and Wilcoxon test using GraphPad5. Graphs presented as pooled data of percentage mean of survival for each genotype. Graphs presented as mean ± SEM.

Oldham et al (2002) reported that viable recessive *Pten* ^*2L117*^/*Pten* ^*2L100*^ mutant combinations, which produce a modest increase in adult weight, are hypersensitive to water-only and starvation stress [30]. We investigated whether the *Pten* ^*5*^ allele, which does not reduce *Pten* function sufficiently to produce consistent *Pten*-dependent effects on growth, also altered the response of adult flies to stresses. The stress assays we employed [27,30,31] typically use males to avoid confounding factors related to female reproductive biology, which may alter the sensitivity of flies to stressors [33]. One to two day-old *Pten* ^*5*^ */Pten* ^*dj189*^ mutant males, *Pten* ^*5*^ */Pten* ^*dj189*^ males carrying a *Pten* genomic rescue construct, and wild type *w* ^*1118*^ control males (the genetic background in which the *Pten* alleles were generated) were exposed to the following stresses at 25°C; 5mM rotenone (an inhibitor of mitochondrial complex 1) (Fig 2A), 2mM paraquat (to induce oxidative stress) [34] (Fig 2B), water-starvation (Fig 2C) and 500mM NaCl (Fig 2D) to increase extracellular salt levels and therefore induce osmotic stress [35]. The number of surviving flies was recorded every 24h.

*Pten* ^*5*^ */Pten* ^*dj189*^ mutant males showed significantly increased susceptibility to paraquat, rotenone, high NaCl and water-only starvation compared to control *w* ^*1118*^ flies. Except in the case of high NaCl stress, these defects were significantly rescued by a *Pten* genomic construct. These results indicate that normal levels of PTEN are protective against a number of different stresses and even modest changes in activity that have no effect on growth in *Pten* ^*5*^ */Pten* ^*dj189*^ males reduce the viability of flies when exposed to such stresses.

### *Pten* ^*5*^ transheterozygous mutants exhibit locomotor phenotypes

We noticed that a small proportion (< 5%) of newly eclosed *Pten* ^*5*^ transheterozygous mutant flies displayed a characteristic outstretched and paralysed wing phenotype. They were unable to fly. We investigated whether other mutant flies might also have a flightless phenotype and whether this phenotype might progress with age using a simple flight assay. Since males and females with altered IIS activity do not always behave similarly in stress and ageing assays [33], we tested both sexes in subsequent experiments. Furthermore, given that there are no characterized *Pten* alleles that are protein nulls, we used multiple *Pten* ^*5*^ transheterozygous combinations to ensure that phenotypes were not caused by an allele-specific interallelic interaction. Although there were some sex-specific and allele-specific differences in the proportions of flies showing a flightless phenotype, almost all effects of genetic modifiers that we tested were independent of the alleles and sex tested.

**Fig 3:**
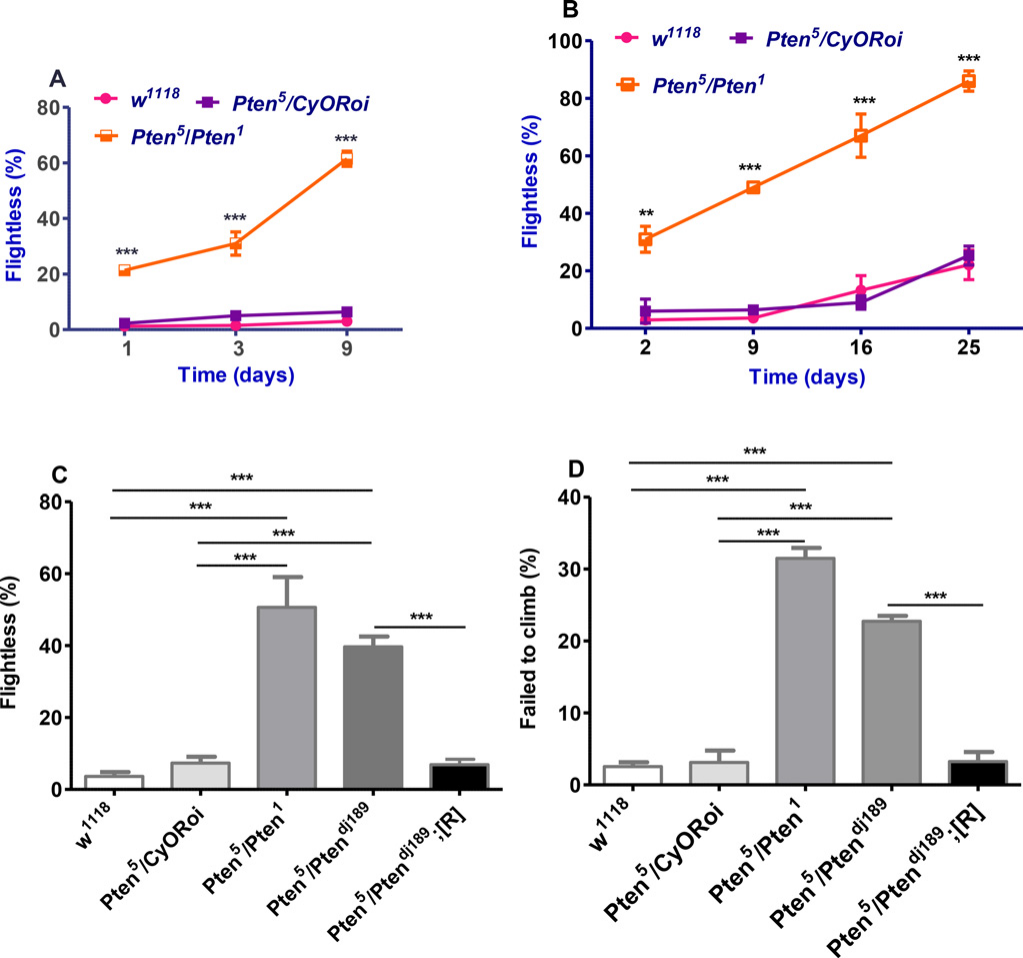
*Pten* ^*5*^ transheterozygous mutants exhibit *Pten*-associated locomotive phenotypes. **(A,B)** Flight tests of adult females of different genotypes over a 9 day **(A)** and 25 day **(B)** period. **(A)** *Pten* ^*5*^ transheterozygous mutant female flightlessness rises between 3 and 9 days (***P<0.001) and is significantly higher than *w* ^*1118*^ and heterozygous *Pten* ^*5*^ */CyORoi* controls over a 9 day period; ***P < 0.001 relative to both controls. There was no statistically significant difference between wild type *w* ^*1118*^ and heterozygous control *Pten* ^*5*^ */CyORoi* at any time point; graphs represent pooled data from six experiments, n ≥ 120. **(B)** Frequency of flightless phenotype for *Pten* ^*5*^ transheterozygous mutant female flies continues to increase (*P* < 0.001 from days 2–9, 9–16 and 16–25 days) and be significantly greater than control *w* ^*1118*^ and *Pten* ^*5*^ */CyORoi* females over a 25 day period. Data from at least six independent experiments. **P < 0.01, ***P < 0.001, n ≥ 100, determined by two-way ANOVA with Bonferroni post-hoc correction for **A** and **B**. **(C)** Flightless phenotype in 9-day-old *Pten* ^5^ transheterozygous female flies is strongly rescued by a *Pten* genomic construct; pooled data from six experiments; *** *P* < 0.001, n ≥ 100. **(D)** 9-day-old *Pten* ^*5*^ transheterozygous mutant males display a defective geotaxic phenotype compared to *w* ^*1118*^ controls, *Pten* ^*5*^ */CyORoi* heterozygotes or genomic rescue flies, assessed by scoring flies that failed to climb 6 cm in 30 sec; n ≥ 50, *** *P* < 0.001. Significance determined by one-way ANOVA with Bonferroni post-hoc correction for **C** and **D**. Graphs present as mean ± SEM.

Surprisingly, *Pten* ^*5*^ */CyORoi* flies, like *w* ^*1118*^ flies, were almost all (~95%) able to fly during the first 9 days of adulthood, despite their curly wings (Fig 3A). For mutant animals, there was a steady rise in the level of flightlessness with age for both female and male mutant flies compared to heterozygous and wild type controls. Over a longer time course, flightlessness for *Pten* ^*5*^ */Pten* ^*1*^ mutant females increased from 31% at day 2 to 86% at day 25, which is significantly higher than *Pten* ^*5*^/*CyORoi* or *w* ^*1118*^ control flies at these time points (both ~ 20%; Fig 3B). A similar progressive flightless phenotype was observed in male *Pten* ^*5*^ */Pten* ^*1*^ transheterozygous mutants (S2A Fig).

From our flight test assays at day 9, between 50–60% of transheterozygous mutant *Pten* ^*5*^ flies failed to fly, while the proportion of flightless flies was not significantly different between *w* ^*1118*^ and *Pten* ^*5*^ */CyORoi* flies at 9 days (less than 10%) or any other age (Fig 3A and 3B and S2A Fig). We used the 9 day time point to determine the effect of manipulating genes involved in the IIS signalling cascade and other signalling pathways, so that interactions that either increased or decreased levels of flightlessness could be detected. To test whether the *Pten* ^*5*^ mutant flightlessness phenotype is caused by loss of *Pten* function, flight activity of *Pten* ^*5*^ */Pten* ^*dj189*^ animals carrying a *Pten* genomic construct was assayed. This mutant combination appeared to have a slightly less penetrant flightless phenotype than *Pten* ^*5*^ */Pten* ^*1*^, as we had found for the eye disorganization phenotype. However, the flightless phenotype was significantly rescued in mutant females (Fig 3C) and males (S2B Fig).

We also investigated whether *Pten* ^*5*^ transheterozygous mutant flies have other motor defects by performing a climbing assay. This assay is typically performed with males, which when tapped to the bottom of a vial normally show geotaxic behaviour and climb rapidly to the top of the vial [36]. Interestingly, at day 9, 30% of *Pten* ^*5*^/*Pten* ^*1*^ and 24% of *Pten* ^*5*^/*Pten* ^*dj189*^ males failed to climb 6 cm within a 30 sec time period, whereas significantly less controls and mutants carrying a genomic rescue construct (about 2% in both cases) showed a similar defect (*P* <0.001; Fig 3D), indicating that the motor defect is caused by *Pten* loss-of-function [29]. Thus, *Pten* ^*5*^ transheterozygous flies have multiple motor defects with flight activity particularly sensitive to reduced *Pten* function. Interestingly, flies lacking the homologues of Parkinson’s disease susceptibility genes PTEN-induced putative kinase 1 (*Pink1*) or *parkin*, which modulate mitochondrial dynamics and function [37], also display motor defects that are most severe in indirect flight muscle [28,31,38,39]. This, together with the progressive nature of the *Pten* ^*5*^ transheterozygous mutant phenotype, raised the possibility that the age-related functional decline in these animals might be linked to a degenerative disorder.

### The flightless phenotype of *Pten* ^*5*^ transheterozygous mutants is associated with muscle-specific defects that involve increased Akt/mTORC1 signalling

To test whether the *Pten* ^*5*^ progressive flightless mutant phenotype might involve cell degeneration, the cell death inhibitor *buffy*, a member of the B cell lymphoma 2 (Bcl-2) family [17], was ubiquitously overexpressed in mutant flies using the *armadillo-* (*arm-*) *GAL4* transcriptional driver. Overexpression of *buffy* can rescue the muscle-specific mitochondrial and degenerative phenotypes seen in flies lacking *pink1*, but does not rescue the flightless defect [28].

**Fig 4:**
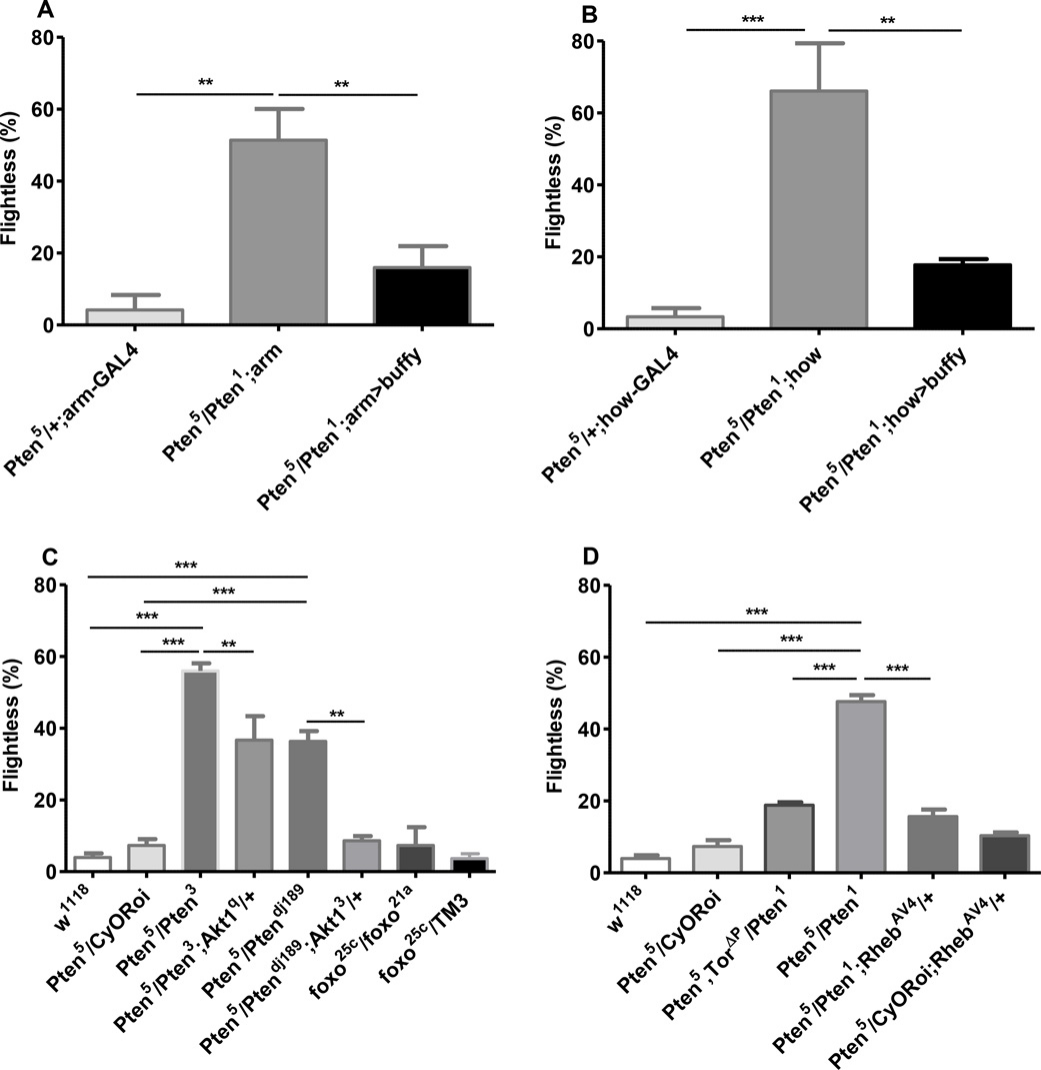
The *Pten* ^*5*^ transheterozygous flightless phenotype involves muscle-specific defects and is suppressed by reducing Akt/mTORC1 signalling. (**A**) Overexpression of the Bcl-2 homologue *buffy* with the ubiquitous *arm-GAL4* driver rescues the *Pten* ^*5*^ */Pten* ^*1*^ transheterozygous mutant flightless phenotype in 9-day-old females; pooled data from nine experiments; **P < 0.005, n ≥50. (**B**) Similarly, overexpression of *buffy* with the *how-GAL4* muscle-specific driver also rescues the *Pten* ^*5*^ */Pten* ^*1*^ flightless phenotype in females; pooled data from six experiments; *** *P* < 0.002, n ≥ 50. (**C**) Reducing IIS through heterozygous loss-of-function *Akt1* ^*q*^ and *Akt1* ^*3*^ alleles significantly suppresses the *Pten* ^*5*^ transheterozygous mutant flightless phenotype in females, while transheterozygous *foxo* ^*25c*^/*foxo* ^*21a*^ null females does not behave significantly differently from *foxo* ^*25c*^/*TM3*, *Pten* ^*5*^ */CyORoi* or *w* ^*1118*^ controls. Data are pooled from at least six experiments. **P < 0.01, n ≥ 70 for *Akt1* ^*q*^, n ≥148 for *Akt1* ^*3*^, n ≥ 80 for *foxo* experiment. (**D**) Reducing mTORC1 signalling in flies heterozygous for the *Tor* ^*ΔP*^ (pooled data from fifteen experiments, n ≥ 139) or *Rheb* ^*AV4*^ (pooled data from four experiments, n ≥ 140) loss-of-function alleles significantly suppresses the *Pten* ^*5*^ */Pten* ^*1*^ transheterozygous flightless phenotype in females; ***P < 0.001. Significance was determined by one-way ANOVA with Bonferroni post-hoc correction test.

Since culture conditions can affect stress responses and lifespan of flies, and *Pten* ^*5*^/*CyORoi* flies behave like *w* ^*1118*^ controls in flight assays, we used *Pten* ^*5*^/*CyORoi* flies as well as *w* ^*1118*^ flies as controls in this and subsequent assays, because the former flies could be selected from the same vials as the *Pten* ^*5*^ transheterozygous mutants that we tested. Adult flies were tested for flight activity at day 9. We observed significant rescue of the *Pten* ^*5*^/*Pten* ^*1*^ flightless phenotype in female (Fig 4A) and male (S3A Fig) flies expressing *buffy* ubiquitously. Furthermore, muscle-specific expression of *buffy* using the muscle-specific *how-GAL4* driver [40] was also sufficient to produce a strong rescue of the progressive flightless defect in females (Fig 4B), though the effect did not reach significance for males (S3B Fig). These data indicate that the *Pten* ^*5*^ recessive mutant flightless phenotype is primarily due to muscle-specific defects. One simple explanation is that *buffy* rescues a muscle degeneration phenotype in these experiments, as it does for *pink1* mutants. However, even 26 day old *Pten* ^*5*^/*Pten* ^*1*^ mutant flies do not display the characteristic indented thoracic phenotype observed in *pink1* flies, suggesting that major indirect flight muscle degeneration has not taken place.

PTEN protein has been reported to function both as a lipid and protein phosphatase [13,41]. Its lipid phosphatase activity antagonises the lipid kinase PI3K, modulating levels of phosphatidylinositol 3,4,5-trisphosphate (PIP3), an activator of the intracellular kinase Akt1 [42]. To test whether increased activation of Akt1 might explain the flightless phenotype in *Pten* ^*5*^ transheterozygotes, we generated mutant flies also carrying one copy of two different recessive *Akt1* alleles to reduce the gene dosage of this target. The effect of the *Akt1* ^*3*^ [22] allele was tested in a *Pten* ^*5*^ */Pten* ^*dj189*^ mutant background, while the *Akt1* ^*q*^ [21] allele was tested in flies carrying *Pten* ^*5*^ and another molecularly uncharacterised genetic null mutation, *Pten* ^*3*^ [13,43], which like *Pten* ^*1*^, gives a more penetrant flightless phenotype than *Pten* ^*dj189*^, which is accentuated in males, when in combination with *Pten* ^*5*^ (Fig 4C and S3C Fig). The flightless phenotype of these two *Pten* ^*5*^ transheterozygous combinations at 9 days was significantly suppressed by the *Akt1* alleles in females (Fig 4C) and males (S3C Fig), indicating that the mutant phenotype is mediated by elevated Akt1 activity.

The transcription factor FOXO is a direct target for Akt1 kinase, which blocks FOXO nuclear translocation, suppressing the expression of multiple genes with roles in combating oxidative stress, amongst other processes [20]. Flight activity of 9-day-old flies carrying a viable, transheterozygous combination of two *foxo* null alleles, *foxo* ^*25c*^ */foxo* ^*21a*^ [19,20] and *foxo* ^*21a*^ heterozygous controls was assayed to test if this gene might be responsible for the *Pten* ^*5*^ phenotype. *foxo* null females and males did not display a significantly increased flightless phenotype (Fig 4C and S3C Fig), indicating that reduced *foxo* activity cannot be the major contributor to the flightless phenotype.

Altered mTORC1 signalling can modulate phenotypes linked to IIS, including degeneration [1,8]. Since mTORC1 is an indirect downstream target of PI3K/Akt, we reasoned that elevated mTORC1 signalling might be responsible for the *Pten* ^*5*^ transheterozygous mutant flightless phenotype. To test this hypothesis, we investigated whether genetic reduction in mTORC1 signalling by altered gene dosage could suppress the flightless phenotype. Akt1 enhances mTORC1 activity via Rheb. There was a significant suppression of the 9-day flightless phenotype in *Pten* ^*5*^/*Pten* ^*1*^ transheterozygotes carrying one copy of the strong loss-of-function allele *Rheb* ^*AV4*^ in females (Fig 4D) and males (S3D Fig). In addition, one copy of the *Tor* ^*ΔP*^ loss-of-function null allele [23] significantly suppressed the *Pten* ^*5*^/*Pten* ^*1*^ flightless phenotype in 9-day-old females (Fig 4D). There also appeared to be suppression in males, but this did not reach significance (S3D Fig). Overall, we conclude that increased mTORC1 signalling is involved in the *Pten* ^*5*^ progressive flightless phenotype and that modest reductions in this signalling can significantly suppress the functional deficits in multiple genetic combinations.

### The *Pten* ^*5*^–dependent transheterozygous flightless phenotype appears to be associated with an oxidative stress response and defects in mitochondrial integrity

**Fig 5:**
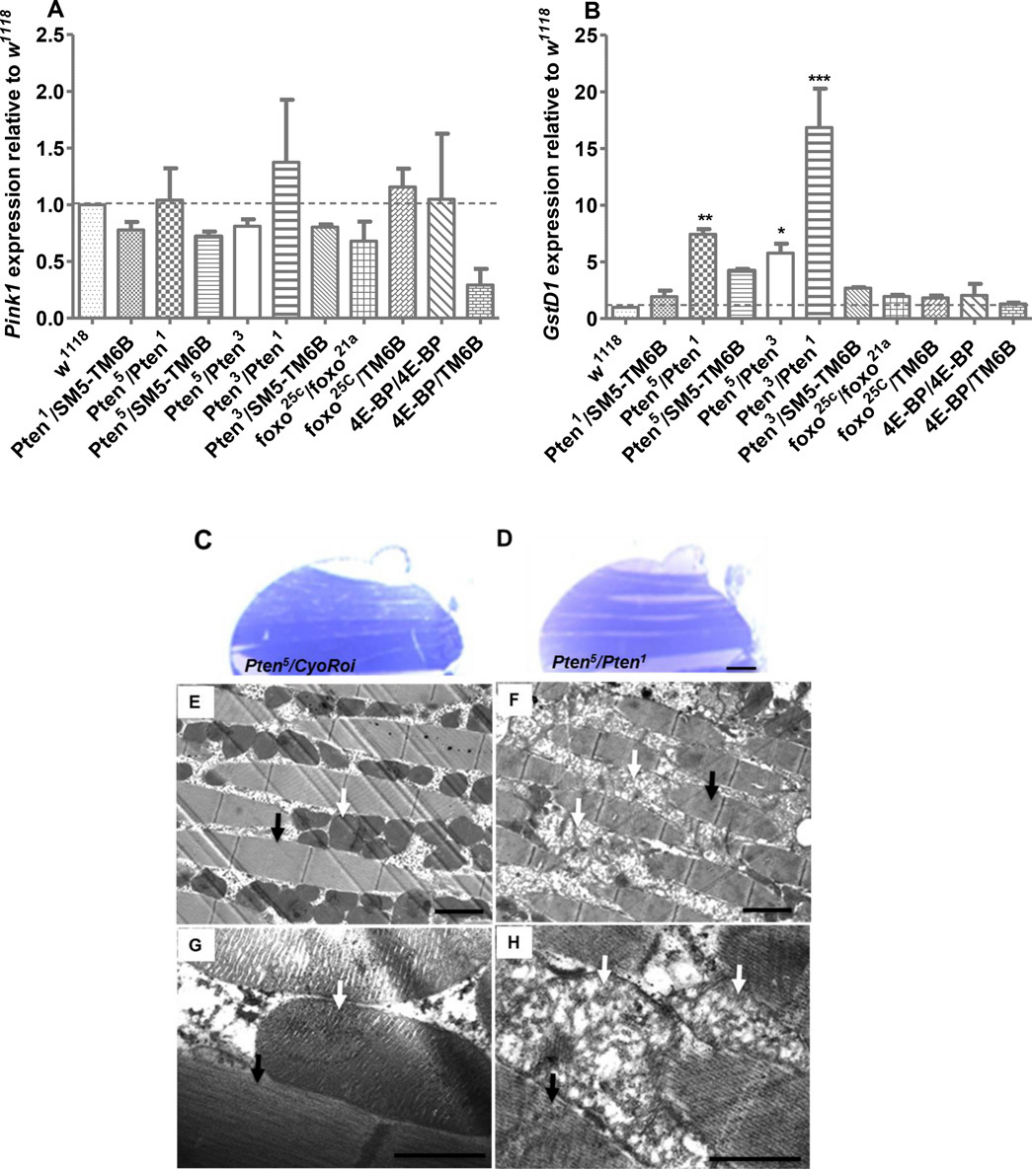
Transheterozygous *Pten* ^*5*^ mutants exhibit defects in mitochondrial structure in IFM and upregulation of the oxidative stress response gene, *GstD1*. (**A**) qRT-PCR of *Pink1* mRNA expression levels in third instar larvae carrying different mutations affecting IIS/mTORC1 signalling normalised to wild type *w* ^*1118*^ control animals. (**B**) Levels of the anti-oxidative enzyme-encoding *GstD1* transcript are elevated significantly in *Pten* mutant backgrounds compared to *w* ^*1118*^ controls. However, there is no significant modulation in the transcript expression levels of *GstD1* in either *foxo* or *4E-BP* mutants or *Pten* heterozygous animals. Data are presented as mean ± SEM. * *P* <0.05; ** *P* < 0.001; ***P < 0.0001, and are from three independent experiments. Significance was determined by one-way ANOVA with Bonferroni post-hoc correction test. (**C**-**H**) Longitudinal sections of thoraces of 26-day-old female flies either stained with toluidine blue and visualized by light microscopy (scale bar: 100μm; **C**,**D**) or imaged by transmission electron microscopy (TEM; scale bar: 1μm) to visualize ultrastructure of IFMs (**E**-**H**). The sarcomeric structure of mutant muscle appears relatively normal (black arrows in **F**,**H** compared to controls in **E**,**G**), but mitochondrial morphology in the mutant is severely disrupted (white arrows in **F**,**H** compared to controls in **E**,**G**).

Mammalian PTEN-induced kinase1 (Pink1) was first identified as a downstream target of *Pten* in cancer cells [44]. Since *Pink1* mutant flies have a flightless phenotype [28,31], we reasoned that the flightless phenotype of *Pten* ^*5*^ transheterozygotes could result from a reduction in *pink1* expression. However, we did not detect any differences in *pink1* transcript levels in adult *Pten* ^*5*^ transheterozygous *Pten* ^*5*^ */Pten* ^*1*^ (1.04 ± 0.28), *Pten* ^*5*^ */Pten* ^*3*^ (0.81 ± 0.06), *Pten* ^*3*^ */Pten* ^*1*^ (1.37±0.5) flies versus wild type *w* ^*1118*^ (normalised to 1.0) controls (*P* >0.06). To investigate this further in genetic backgrounds where IIS/mTORC1 signalling is more severely reduced, *pink1* transcript levels were measured using qRT-PCR in mutant third instar larvae in which different components of the IIS/mTORC1 pathway, including *Pten*, the IRS1-4 homologue (*chico*), *foxo* and the mTORC1 target and translational regulator *4E-BP* (*Thor*) were strongly reduced. *w* ^*1118*^ flies were used as controls. Expression levels were normalised to the ribosomal housekeeping gene *RpL32*. There was no significant change in *pink1* mRNA in any of the IIS/mTORC1-modulated genetic backgrounds, suggesting that this pathway does not control *pink1* levels, at least during larval development (Fig 5A).

Studies in mammalian systems have indicated that the transcription factor nuclear factor erythroid-derived 2-like 2 (Nrf-2) can be activated via the hyperactivation of the PI3-kinase/Akt signalling cassette to regulate genes involved in oxidative stress response [45]. Given that *Pten* ^*5*^ transheterozygous animals appear to have hyperactivated IIS and PI3-kinase/Akt1 signalling, we reasoned that key targets of Nrf2, which include glutathione S-transferase D1 (GstD1), might be significantly altered in *Pten* ^*5*^ transheterozygotes, despite the increased sensitivity to oxidative stress, while other genes directly regulating mitochondrial function might not be differently expressed.

We analysed expression of mitochondrial transcription factor A (TFAM), mitochondrial transcription factor B2 (*mtTFB2*), and the Nrf1 family transcription factor, *erect wing* (*ewg*), which are all involved in expression of mitochondrial genes. They were unaffected in larvae with defective IIS/mTORC1 signalling (S4A Fig). However, there was a significant elevation in transcripts encoding GstD1 in all *Pten* mutant combinations, including not only the strongest allelic combination of *Pten* ^*3*^ */Pten* ^*1*^, which rarely produces adult flies, but also combinations with the *Pten* ^*5*^ allele (Fig 5B). We conclude that even relatively subtle changes in IIS signalling can upregulate expression of *GstD1*. However, *Pten* ^*5*^ mutants are still hypersensitive to oxidative stress.

To determine whether the *Pten* ^*5*^ progressive flightless phenotype is associated with any structural or cell degeneration defects in indirect flight muscle (IFM) that could be linked to stress sensitivity, the thoraces of 26-day-old adult *Pten* ^*5*^ transheterozygous mutant females and heterozygous controls were dissected. Longitudinal sections were visualised by light microscopy and transmission electron microscopy (TEM). No obvious differences were seen between the IFMs of control (Fig 5C) and mutant (Fig 5D) animals by light microscopy in toluidine blue-stained sections, confirming that no major tissue degeneration was present. Under TEM, in sharp contrast to the defects seen in *pink1* mutant flies [28,31], the sarcomeric structure and muscle fibre organisation in mutants seemed relatively normal. However, like *pink1* mutants, there was a drastic disruption of mitochondrial morphology in IFMs (Fig 5F and 5H) compared to controls (Fig 5E and 5G). These findings indicate that reduced *Pten* activity leads to disruption of mitochondrial integrity, which presumably contributes to the flightless phenotype seen in most *Pten* ^*5*^ mutant animals at 26 days.

## Discussion

In this report, we describe a new hypomorphic viable recessive mutation in the IIS antagonist *Pten*, which is associated with increased stress sensitivity, a progressive adult flightless phenotype and severe mitochondrial disruption within the indirect flight muscles in aging flies. We use genetic approaches to show that the flightless phenotype is dependent on increased IIS and mTORC1 signalling, and demonstrate that even though there is no major degeneration of flight muscle, the phenotype can be rescued by muscle-specific expression of the *Bcl-2* family member, *buffy*. We conclude that subtle increases in IIS are sufficient to induce mitochondrial disruption and functional decline in highly metabolically active tissues like IFMs.

### Modest reductions in *Pten* activity induce IIS/mTORC1-dependent progressive functional decline in indirect flight muscle

In contrast to a previously identified viable hypomorphic allele of *Pten* [30], *Pten* ^5^ transheterozygous mutants have no consistent growth defects relative to control flies, suggesting that this allele only subtly reduces *Pten* function. We did observe modest weight differences for specific genotypes, but these effects were allele- and sex-specific. Since none of the *Pten* alleles we employed are likely to be protein nulls, we reasoned that phenotypes could only be assigned to loss of *Pten* function if they were observed with multiple interallelic combinations and could be rescued with a *Pten* genomic construct. We found that *Pten* ^*5*^ mutant flies do exhibit a number of other adult phenotypes, including sensitivity to several stresses, and age-dependent locomotor defects that fulfill these criteria, strongly indicating that they are caused by reduced *Pten* function.

*Pten* ^5^ transheterozygous mutant animals exhibit a characteristic subtle disruption of the ommatidia in the posterior region of the eye. The eye phenotype, which is nearly 100% penetrant, is presumably a developmental defect associated with differentiation or subsequent reorganization of cells during pupal development. It proved useful in our analysis as a marker for presence of the *Pten* ^*5*^ transheterozygous combination in flies where the flightless phenotype, but not the eye defect, was strongly rescued by specific genetic manipulations, such as reduction in *Akt1* or *Tor* activities.

*Pten* ^5^ transheterozygous mutant flies were short-lived when exposed to a range of stresses including paraquat and rotenone. These oxidative stress-inducing agents decrease mitochondrial function by reducing mitochondrial complex I activity and can compromise cell viability [46,47]. Misregulation of mitochondrial activity is also believed to be strongly associated with malfunction of the proteasome which is linked to decline in dopaminergic (DA) neurons as observed in Parkinson’s disease [47]. Reduced life span has previously been observed when other viable *Pten* mutant flies were exposed to altered osmotic conditions and water-only starvation [30], supporting the hypothesis that elevated PI3K/Akt signalling affects protective cellular responses to a wide range of stresses [2].

The progressive flightless phenotype in *Pten* ^*5*^ transheterozygous mutant flies has not previously been observed in other IIS mutants, but is also consistent with an increase in sensitivity to cellular stress in response to elevated IIS. Insect indirect flight muscle is one of the most metabolically active tissues in the animal kingdom [48], requiring high levels of energy and mitochondrial activity. Hence, even a partial defect in mitochondrial function could lead to a severe energy deficiency, and block the contractile function of these cells. Although all *Pten* ^*5*^ transheterozygous flies displayed a progressive flightless phenotype, there were differences in the penetrance of the flightless phenotype between different allelic combinations, presumably reflecting differences in the alleles employed. In addition, there were differences in penetrance and levels of genetic suppression between males and females. There is not an obvious explanation for this, but similar observations have been made for other mutants that affect IIS [25,33], and in many other IIS studies, the data for only one sex are frequently reported.

Importantly, several pieces of evidence strongly support our conclusion that increased IIS/mTORC1 signalling causes the flightless phenotype. Using several different *Pten* ^*5*^ allelic combinations, we showed that reducing *Akt1*, *Rheb* and *Tor* gene dosage significantly suppresses the phenotype. Furthermore, molecular characterisation of the *Pten* ^*5*^ allele indicates that the mutant protein carries a mutation in a conserved residue of the lipid phosphatase TI-loop. Based on studies of the human protein [32], this would be predicted to reduce but not completely block PTEN’s IIS regulatory activity.

It was not possible to test whether increasing IIS specifically in muscle was responsible for the flightless phenotype, because we could not express IIS antagonists like PTEN with muscle drivers at sufficiently low levels to avoid lethality due to developmental defects. But, muscle-specific expression of *buffy* [17,49] strongly protected against age-related functional decline in females, while its effects did not reach significance in males, suggesting that the phenotype primarily involves defects in muscle tissue. However, we cannot completely eliminate the possibility that defects in other tissues, such as the nervous system, are also involved.

Since about 20–30% of *Pten* ^*5*^ transheterozygotes are unable to fly shortly after eclosion, it is also conceivable that the *Pten* ^*5*^ mutation causes a developmental defect that is responsible for functional defects that appear many weeks later in adults. Adult-specific *Pten* rescue will be necessary to properly demonstrate that this is not the case. However, the fact that mutant adults are more susceptible to multiple stresses, including mitochondrial inhibitors, argues that post-developmental defects are present that could provide the trigger for the emergence of functional deficits in ageing adult muscle.

On the basis of the climbing defects observed in mutant flies, it appears that the functions of other muscles are also altered by the *Pten* ^*5*^ allele. However, we did not undertake a detailed investigation of this phenotype, because there is more variability in the relatively small proportion of flies exhibiting this phenotype in standard assays, making it difficult to score partial rescue activity reliably.

Although genetically decreasing IIS-mTORC1 signalling in *Pten* ^*5*^ transheterozygous mutants by reducing dosage of *Rheb*, *Tor*, and *Akt1* showed a consistent suppression of the flightless phenotype, complete loss-of-function for *foxo* did not produce a significant increase in flightlessness compared to controls, even though reduced *foxo* activity in flies and other organisms induces some forms of stress sensitivity and can also affect growth, though only when IIS is decreased [19,20,50]. These findings indicate that multiple downstream targets of Akt1 have roles in controlling stress sensitivity, but that the mTORC1 pathway is critical in IIS-induced, age-dependent functional decline in IFMs under normal culture conditions.

### Reduced *Pten* activity induces elevated levels of oxidative stress response and defects in mitochondrial integrity

Mitochondria in the IFMs of *Pten* ^*5*^ transheterozygous mutants cultured for 26 days had severely disrupted morphology, unlike control animals cultured for the same period of time. This phenotype is reminiscent of the effects of loss-of-function mutants for the two Parkinson’s disease susceptibility genes, *Pink1* and *Parkin* [29,31], which also have selective effects on mitochondria in IFMs and a flightless phenotype [28,31].

Pink1 was first identified as a PTEN-inducible kinase in *Pten* mutant tumour cells transfected with a *Pten* expression construct [44], leading us to hypothesise that the effects of *Pten* ^*5*^ might be mediated by modulation of *Pink1* levels. However, when we measured *Pink1* expression both in *Pten* ^*5*^ transheterozygous animals and in strong *Pten* loss-of-function mutant larvae, which die mostly before pupation, we did not observe significant changes in transcript levels.

The potential similarity between the *Pten* ^*5*^ and *pink1* mutant phenotypes led us to test the effects of muscle-specific *buffy* expression, which rescues the mitochondrial and degeneration defects in *pink1* mutants, but not the flightless phenotype [28]. Although the flightless phenotype could be rescued by *buffy*, subsequent analysis indicated that despite extensive mitochondrial defects, there were no obvious indications of major tissue loss or muscle fibre disorganisation in *Pten* ^*5*^ mutant IFM, in stark contrast to *pink1* mutants. Although Buffy protein has been reported to normally be located on the endoplasmic reticulum [51], it can rescue non-degenerative mitochondrial integrity defects in neurons [52]. Buffy can also suppress basal mTORC1 signalling [53], providing another possible explanation for our data. We conclude that subtly elevated IIS in *Pten* ^*5*^ mutants leads to age-dependent functional deficits in IFM, which include loss of mitochondrial integrity, but do not over the time course of our study induce major cell degeneration.

mTORC1 signalling has been implicated previously as a modulator of degenerative processes in experiments that either employ strong overexpression of *Rheb* in muscle [11] or reduced mTORC1 signalling in Huntington's and Alzheimer's models [54]. Whether the *Pten* ^*5*^ IFM phenotype reflects the early stages of such degenerative processes or the inability to combat cellular events that precede cell death in a sensitive tissue like muscle [29,55] remains unclear. In one possible example of the latter mechanism, autophagy, which is suppressed by IIS, is critical in combating damage in many cellular systems, including that caused by increased ROS production [55], thus maintaining mitochondrial integrity and function under normal conditions.

Studies in animal models have indicated that oxidative stress due to increased levels of reactive oxygen species (ROS) can initiate degenerative events and disrupt mitochondria [46]. Upregulation of oxidative stress response pathways is a key cytoprotective mechanism by which cells detoxify ROS [7]. Several anti-oxidative enzymes have been shown to be upregulated in response to ROS from mouse to *Drosophila* [56]. Interestingly, *GstD1* was significantly altered in *Pten* ^*5*^ and other *Pten* mutants, but not in *foxo* and *4E-BP* mutant animals. These data suggest that although *Pten* ^*5*^ is a weak hypomorphic *Pten* allele, it is still capable of inducing oxidative stress response genes, but presumably not to a level that provides normal protection against agents that induce oxidative stress like paraquat. Many studies have shown that increased production of ROS inevitably diminishes mitochondrial ATP production and consequently leads to increased levels of misfolded proteins and intracellular aggregates, which will accumulate more rapidly if autophagy is suppressed. This inadequate protection may explain why IFM mitochondria and physiological function are not maintained normally during ageing.

It is also important to consider more direct effects of PTEN on mitochondrial function in explaining the *Pten* ^*5*^ phenotype. In mammals, different forms of PTEN localise to mitochondrial membranes and can modulate metabolism and apoptotic signalling [57–59], and this may play a part in the phenotypes we observe. However, some of these effects in mammals seem to be independent of phosphatase activity; these are probably not involved, because they would be unlikely to be suppressed by modest reduction of IIS as we see in flies.

In summary, this work demonstrates for the first time that in addition to raising sensitivity to cellular stresses, subtle increases in IIS are sufficient to induce functional decline in indirect flight muscle, which is associated with mitochondrial disruption in *Pten* ^*5*^ mutants. These animals show an elevated oxidative stress response, but ultimately the metabolically active cells of the IFM fail to maintain normal cellular structure. Although this tissue is also particularly susceptible to mitochondrial loss and degeneration in flies mutant for genes associated with Parkinson’s disease, it remains unclear whether these phenotypes are linked. However, our findings using mutants which subtly reduce IIS/mTORC1 signalling to suppress the *Pten* ^*5*^ phenotype suggest drug treatments that only modestly decrease mTORC1 signalling may be beneficial in treating some forms of age-related disease where IIS is defective.

## Supporting Information

S1 Fig Transheterozygous *Pten* ^*5*^ mutant male flies exhibit a highly penetrant eye phenotype, but body mass is not consistently affected. (**A**) A mild disorganization of the ommatidia in the posterior region of the eye was observed in *Pten* ^*5*^ */Pten* ^*1*^ and *Pten* ^*5*^ */Pten* ^*dj189*^ male flies. This eye phenotype is completely absent in wild type *CantonS* and heterozygote *Pten* ^*5*^ */CyO* control males and completely rescued in *Pten* genomic rescue males. Data are presented as mean percentage of flies exhibiting disorganised eye phenotype; *** *P <* 0.001. Data from two independent experiments, n ≥100. (**B**) The mean body masses of different *Pten* mutant males. *Pten* ^*5*^ */Pten* ^*1*^, and *Pten* genomic rescue *Pten* ^*5*^ */Pten* ^*dj189*^ mutant males have a significantly greater mass than *w* ^*1118*^ and *Pten* ^*5*^ */CyORoi* control males, but the rescue males do not have a reduced mass relative to mutants. Data from two independent experiments, n ≥ 50. Pooled data presented as mean body mass per fly ± SEM. Significance was determined by two-tailed unpaired Student’s *t*-test.
(TIF)

S2 Fig *Pten* ^*5*^ transheterozygous mutant males develop a *Pten*-associated flightless phenotype. (**A**) *Pten* ^*5*^ */Pten* ^*1*^ transheterozygous males exhibit an early-onset progressive (*P* <0.001 for changes between days 2–9, 9–16 and 16–25) flightless phenotype, which was significantly higher at all time points compared with *w* ^*1118*^ and *Pten* ^*5*^/*CyORoi* controls. Pooled data from at least six independent experiments, n ≥ 80; **P < 0.01 and ***P <0.001. (**B**) Flightlessness in *Pten* ^5^ transheterozygous mutant males is rescued by a *Pten* genomic construct. Histogram shows mean percentage of flightless males at day 9 for different genotypes, pooled data of at least six experiments; n ≥100; *** *P* < 0.001. Data are presented as mean ± SEM. Significance was determined by one-way ANOVA with Bonferroni post-hoc correction test.
(TIF)

S3 Fig The *Pten* ^*5*^ transheterozygous flightless phenotype is suppressed by reducing Akt/mTORC1 signalling. (**A**) Overexpression of the Bcl-2 homologue *buffy* with the ubiquitous armadillo (arm) driver, *arm-GAL4*, partially rescues the *Pten* ^*5*^ */Pten* ^*1*^ transheterozygous mutant flightless phenotype in 9-day-old male flies; data from two independent experiments, n = 100. (**B**) Overexpression of *buffy* with the *how* ^*24B*^ *-GAL4* muscle-specific driver does not significantly rescue the phenotype, n = 130. (**C**) Downregulation of IIS induced by heterozygous loss-of-function *Akt1* ^*q*^ and *Akt1* ^*3*^ alleles significantly suppresses the *Pten* ^*5*^ flightless phenotype in males; duplicate experiments, n ≥160. Transheterozygous *foxo* ^*25c*^/*foxo* ^*21a*^ mutants were also analysed together with control transheterozygous *foxo* ^*25c*^/*TM3* control, but they do not significantly induce flightlessness. (**D**) The heterozygous loss-of-function *Rheb* ^*AV4*^ allele, but not *Tor* ^*ΔP*^, significantly suppresses the *Pten* ^*5*^ flightless phenotype in males. Pooled data from three independent experiments, n >110. Data are presented as mean ± SEM. Significance was determined by one way ANOVA with Bonferroni post-hoc correction test. * *P* < 0.05,**P < 0.01, *** *P <* 0.001.
(TIF)

S4 Fig Analysis of mitochondria-associated transcription factor mRNA expression in *Pten* and *foxo* mutant larvae. qRT-PCR of *mitochondrial transcription factor A* (*TFAM-A*, mtTF1, TFAM, *d-TFAM*, *CG4217*), *mitochondrial transcription factor B2 (TFAM-B2*, *d-mtTFB2*, *CG3910)* and Nrf1 family transcription factor/co-activator *erect wing* (*ewg*, *CG3114*) mRNA expression levels in third instar larvae was assessed. mRNA levels in flies carrying different mutations affecting IIS/mTORC1 signalling were normalised to wild type *w* ^*1118*^ control animals to check for altered expression of mitochondria-associated transcription factors in *Pten* and *foxo* mutant animals. Results show pooled data from three independent experiments. The levels of these nuclear transcription factor transcripts were not significantly elevated in either *Pten* or *foxo* mutant backgrounds.
(TIF)
