# Supplementary figures and images for: Fine-Tuning of PI3K/AKT Signalling by the Tumour Suppressor PTEN Is Required for Maintenance of Flight Muscle Function and Mitochondrial Integrity in Ageing Adult Drosophila melanogaster
Source: PLoS One. 2015 Nov 23;10(11):e0143818. doi: 10.1371/journal.pone.0143818 (PMC4658134; doi:10.1371/journal.pone.0143818)

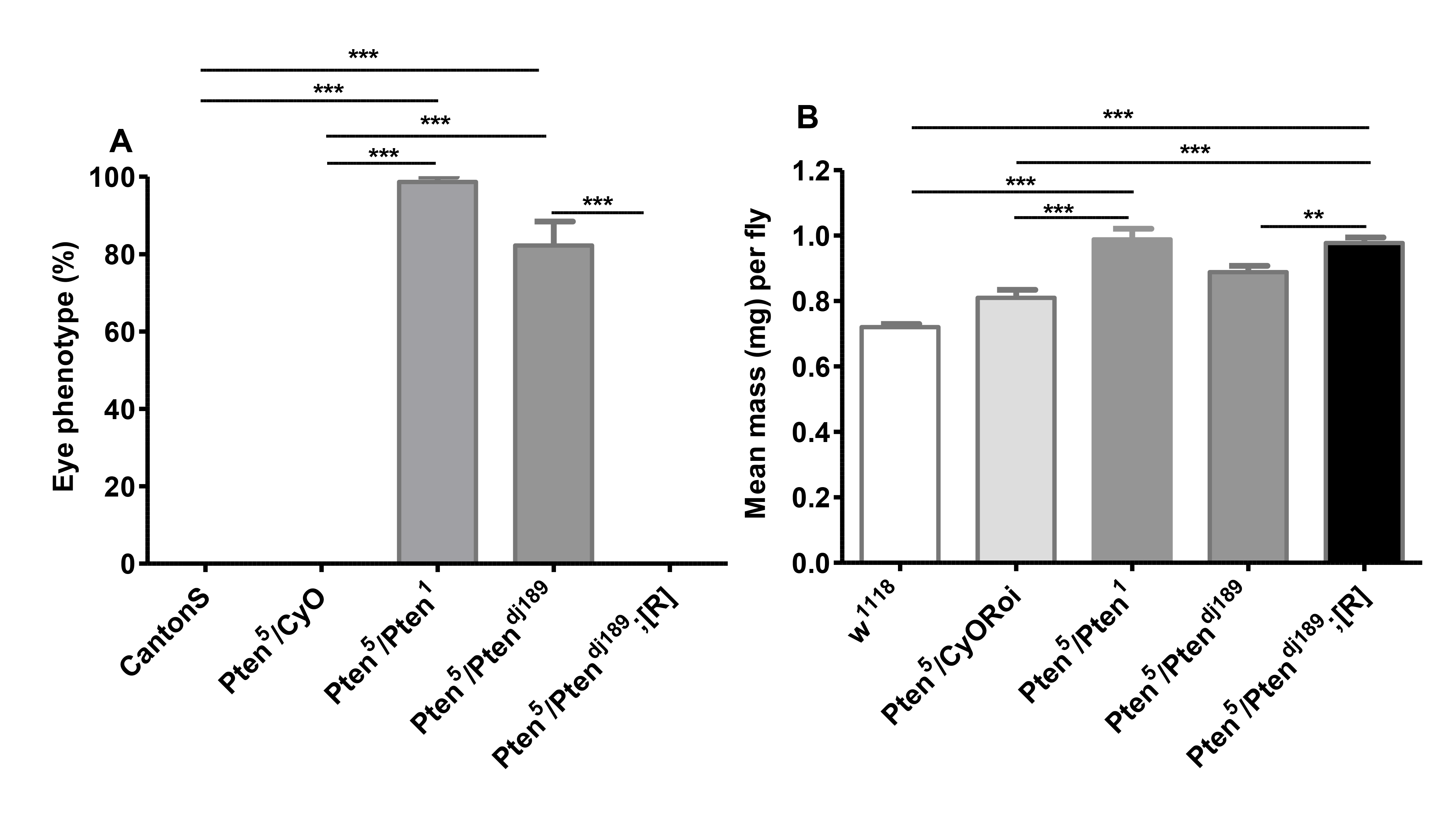

Supplement: S1 Fig — (A) A mild disorganization of the ommatidia in the posterior region of the eye was observed in Pten 5 /Pten 1 and Pten 5 /Pten dj189 male flies. This eye phenotype is completely absent in wild type CantonS and heterozygote Pten 5 /CyO control males and completely rescued in Pten genomic rescue males. Data are presented as mean percentage of flies exhibiting disorganised eye phenotype; *** P < 0.001. Data from two independent experiments, n ≥100. (B) The mean body masses of different Pten mutant males. Pten 5 /Pten 1, and Pten genomic rescue Pten 5 /Pten dj189 mutant males have a significantly greater mass than w 1118 and Pten 5 /CyORoi control males, but the rescue males do not have a reduced mass relative to mutants. Data from two independent experiments, n ≥ 50. Pooled data presented as mean body mass per fly ± SEM. Significance was determined by two-tailed unpaired Student’s t-test. (TIF) [file pone.0143818.s001.tif]

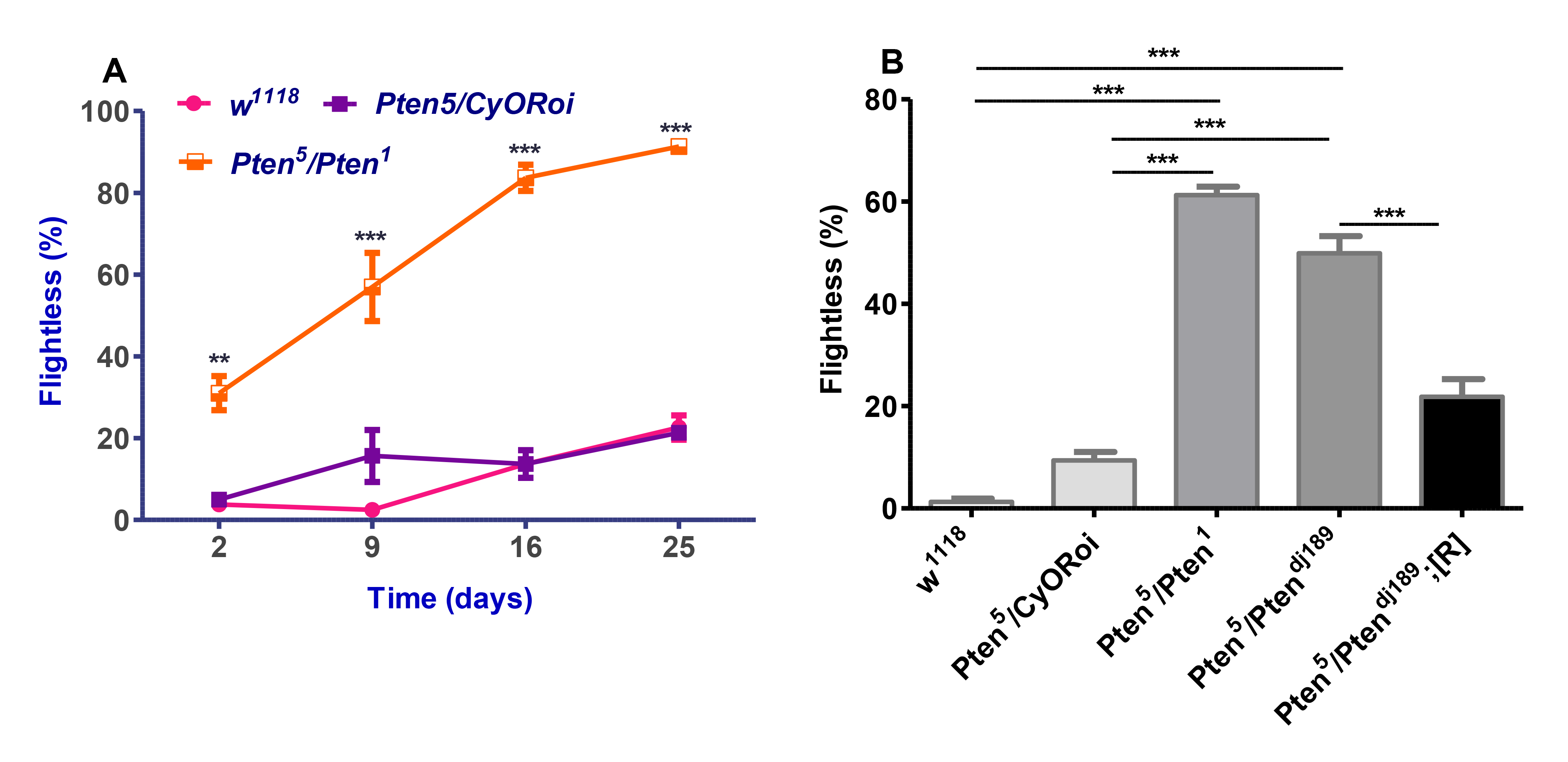

Supplement: S2 Fig — (A) Pten 5 /Pten 1 transheterozygous males exhibit an early-onset progressive (P <0.001 for changes between days 2–9, 9–16 and 16–25) flightless phenotype, which was significantly higher at all time points compared with w 1118 and Pten 5/CyORoi controls. Pooled data from at least six independent experiments, n ≥ 80; **P < 0.01 and ***P <0.001. (B) Flightlessness in Pten 5 transheterozygous mutant males is rescued by a Pten genomic construct. Histogram shows mean percentage of flightless males at day 9 for different genotypes, pooled data of at least six experiments; n ≥100; *** P < 0.001. Data are presented as mean ± SEM. Significance was determined by one-way ANOVA with Bonferroni post-hoc correction test. (TIF) [file pone.0143818.s002.tif]

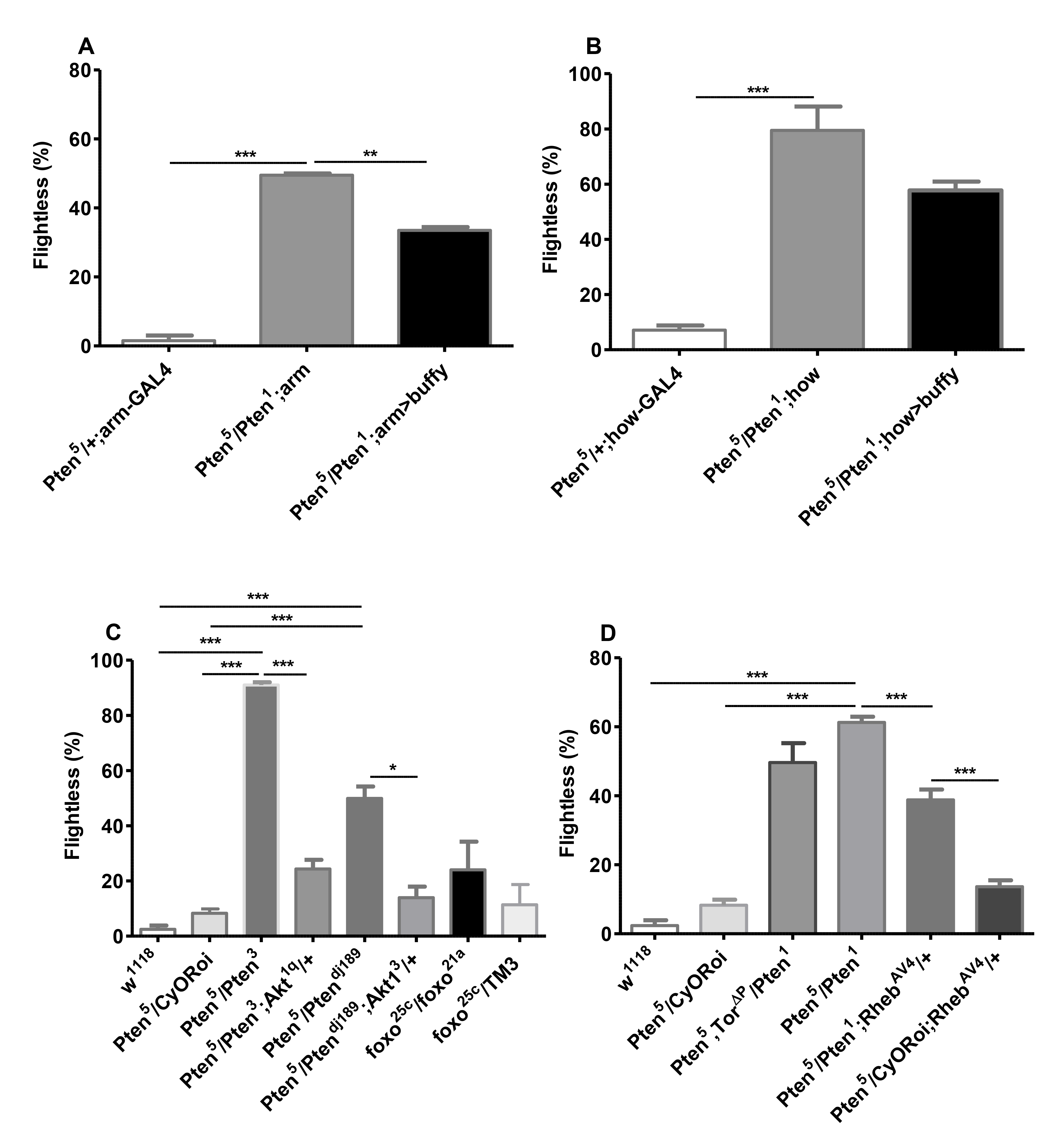

Supplement: S3 Fig — (A) Overexpression of the Bcl-2 homologue buffy with the ubiquitous armadillo (arm) driver, arm-GAL4, partially rescues the Pten 5 /Pten 1 transheterozygous mutant flightless phenotype in 9-day-old male flies; data from two independent experiments, n = 100. (B) Overexpression of buffy with the how 24B -GAL4 muscle-specific driver does not significantly rescue the phenotype, n = 130. (C) Downregulation of IIS induced by heterozygous loss-of-function Akt1 q and Akt1 3 alleles significantly suppresses the Pten 5 flightless phenotype in males; duplicate experiments, n ≥160. Transheterozygous foxo 25c/foxo 21a mutants were also analysed together with control transheterozygous foxo 25c/TM3 control, but they do not significantly induce flightlessness. (D) The heterozygous loss-of-function Rheb AV4 allele, but not Tor ΔP, significantly suppresses the Pten 5 flightless phenotype in males. Pooled data from three independent experiments, n >110. Data are presented as mean ± SEM. Significance was determined by one way ANOVA with Bonferroni post-hoc correction test. * P < 0.05,**P < 0.01, *** P < 0.001. (TIF) [file pone.0143818.s003.tif]

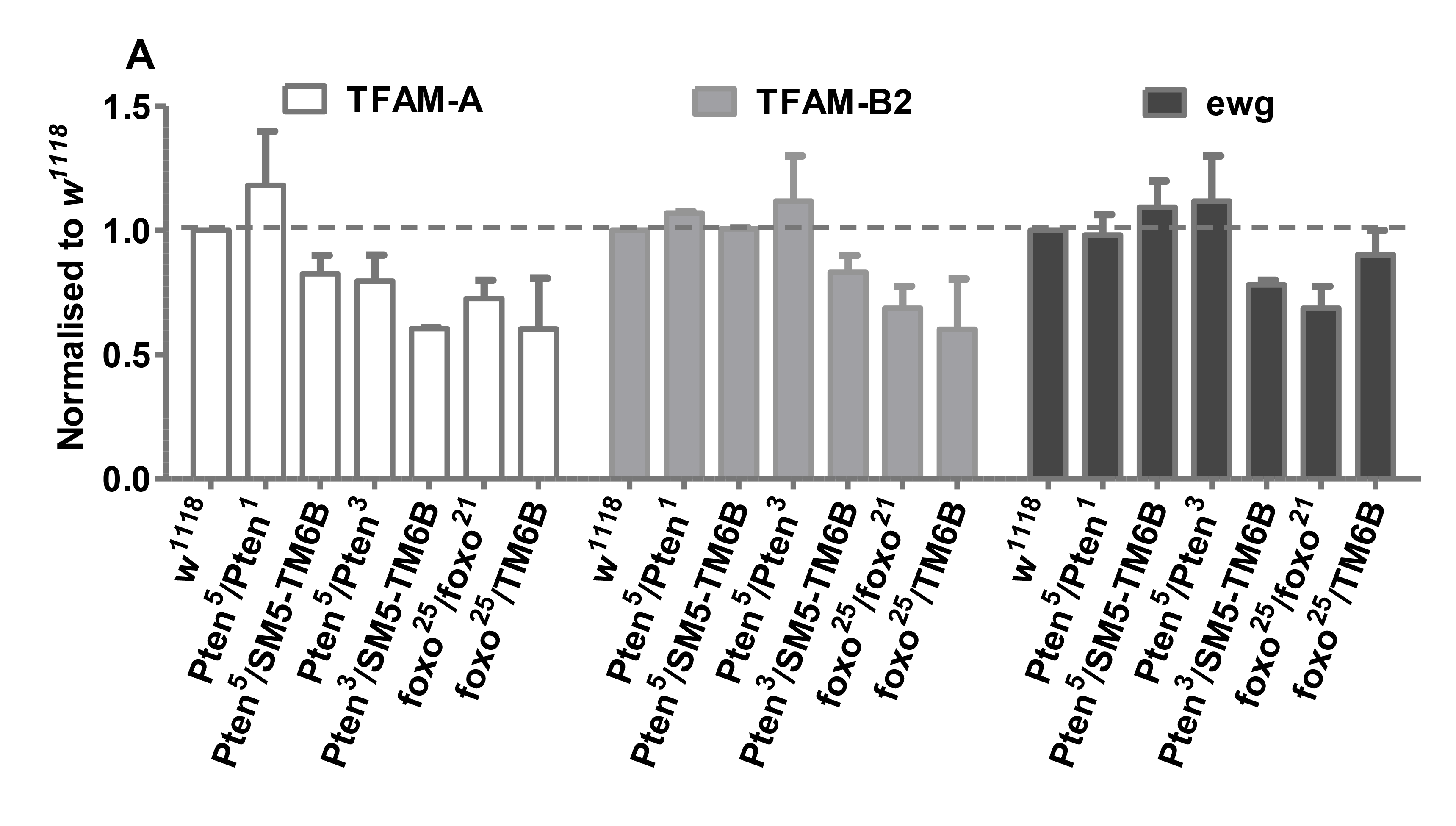

Supplement: S4 Fig — qRT-PCR of mitochondrial transcription factor A (TFAM-A, mtTF1, TFAM, d-TFAM, CG4217), mitochondrial transcription factor B2 (TFAM-B2, d-mtTFB2, CG3910) and Nrf1 family transcription factor/co-activator erect wing (ewg, CG3114) mRNA expression levels in third instar larvae was assessed. mRNA levels in flies carrying different mutations affecting IIS/mTORC1 signalling were normalised to wild type w 1118 control animals to check for altered expression of mitochondria-associated transcription factors in Pten and foxo mutant animals. Results show pooled data from three independent experiments. The levels of these nuclear transcription factor transcripts were not significantly elevated in either Pten or foxo mutant backgrounds. (TIF) [file pone.0143818.s004.tif]
